# Increased Risk of Recurrent Ischemic Stroke in Male Patients Taking Medications for Benign Prostatic Hyperplasia

**DOI:** 10.3390/life16020311

**Published:** 2026-02-11

**Authors:** Chun-Gu Cheng, Chun-Fang Chen, Wu-Chien Chien, Chia-Chao Wu, Hung-Wen Chiu, Fei-Hung Hung, Hung-Pin Peng, Chi-Hsiang Chung, Chun-An Cheng

**Affiliations:** 1 Department of Emergency Medicine, Taoyuan Armed Forces General Hospital, Taoyuan 32549, Taiwan; doc50015@yahoo.com.tw; 2 Department of Emergency Medicine, College of Medicine, Tri-Service General Hospital, National Défense Medical University, Taipei 11490, Taiwan; 3 Department of Emergency Medicine, Cheng Hsin General Hospital, Taipei 11220, Taiwan; victor433628.a@gmail.com; 4 School of Public Health, National Défense Medical University, Taipei 11490, Taiwan; chienwu@mail.ndmctsgh.edu.tw (W.-C.C.);; 5 Department of Medical Research, Tri-Service General Hospital, Taipei 11490, Taiwan; 6 Department of Nephrology, College of Medicine, Tri-Service General Hospital, National Défense Medical University, Taipei 11490, Taiwan; 7 Graduate Institute of Biomedical Informatics, Taipei Medical University, Taipei 11031, Taiwan; 8 Health Data Analytics and Statistics Centre, Office of Data Science, Taipei Medical University, New Taipei City 235041, Taiwan; 9 Research Center of Data Science on Healthcare Industry, College of Management, Taipei Medical University, New Taipei City 235041, Taiwan; 10 Clinical Data Center, Office of Data Science, Taipei Medical University, New Taipei City 235041, Taiwan; 11 Department of Neurology, College of Medicine, Tri-Service General Hospital, National Défense Medical University, Taipei 11490, Taiwan

**Keywords:** recurrent ischemic stroke, benign prostatic hyperplasia, alpha-1 adrenergic blocker

## Abstract

Background: Patients with benign prostatic hyperplasia (BPH) have an increased risk of developing cardiovascular disease. Taking alpha-1 blockers is associated with an increased risk of major adverse cardiovascular events. Patients suffering from ischemic stroke (IS) may develop baroreflex and parasympathetic dysfunction-induced cerebral autoregulation impairment. The relationship between pharmacotherapy for BPH and the risk of recurrent IS remains unclear. The purpose of this study was to determine whether medications for BPH increase the risk of recurrent IS. Methods: This is a retrospective cohort study. Data from patients diagnosed with IS between 2000 and 2015 was collected from Taiwan National Health Insurance Database. Newly diagnosed IS patients were identified (International Classification of Diseases, Ninth Edition, Clinical Modification (ICD-9-CM): 433–437). BPH patients with an ICD-9-CM of 600 were identified. The event observed was recurrent IS after the firstever IS. The factors associated with recurrent IS were assessed via Cox proportional hazards regression. Results: Recurrent IS was associated with BPH with an adjusted hazard ratio (HR) of 1.505 and a 95% confidence interval (CI) of 1.112–1.829, *p* < 0.001), and a competing risk model showed an adjusted HR of 1.544 (95% CI: 1.128–1.896, *p* < 0.001). The adjusted HR for treatment with alpha-1 blockers was 1.581 (95% CI: 1.16–1.915, *p* < 0.001), and increased risk with adjusted HR for treatment with high doses of 5-alpha reductase inhibitors over a long period of time are also at risk of recurrent IS. Conclusions: These findings highlight the association between BPH incidence and the risk of recurrent IS. The pharmacotherapy for BPH in IS patients should take great care.

## 1. Introduction

Benign prostatic hyperplasia (BPH) is a difficult urinary disease in older males caused by nonmalignant enlargement of the prostate, and it can lead to a variety of urinary symptoms and complications. Fifty percent of males over 50 yearsold experience symptoms of BPH [1]. Emerging evidence suggests that patients with BPH often have many cardiovascular risk factors, including hypertension, diabetes, and atherosclerosis [2,3]. Given this overlap, the potential for BPH to cause adverse cardiovascular events has attracted considerable attention in recent years.

Ischemic stroke (IS) is a leading cause of morbidity and mortality worldwide, and its recurrence rate is high, especially in patients with multiple comorbidities. The goal of secondary prevention is to reduce recurrent IS. Although the risk factors for IS disease are strictly controlled, recurrent IS still occurs, suggesting the presence of additional unrecognized risk factors [4]. Patients with IS have reduced cerebral autoregulation function and baroreflex sensitivity to parasympathetic function [5,6]. The risk of stroke in patients with BPH in a Japanese population was reported to be increased, with a hazard ratio (HR) of 1.3 [7]; the incidence of IS was reported to be increased following the initiation of α-blocker therapy (incidence rate ratio 1.4) [8]. Moreover, patients with coexisting BPH and IS have been shown to have a higher risk of recurrent IS within one year (HR 1.35) [9]. Management of BPH in patients with a history of IS is therefore clinically challenging, as commonly prescribed medications that may influence cerebrovascular risk need to be explored.

Alpha-1 adrenergic receptor blockers (alpha-1 blockers) are typically used to treat BPH by relaxing the smooth muscles of the prostate and bladder neck [10,11]. The vasodilation and lowering of blood pressure, as a result of treatment withalpha-1 blockers, raise concerns about potential effects on cerebral blood flow and autoregulation, particularly in patients with compromised vascular integrity, who have an increased risk of experiencing major adverse cardiovascular events (MACEs), with an 8% increased risk compared with patients treated with 5α-reductase inhibitors in the U.S. population [12]. 5α-reductase inhibitors mainly reduce prostate size. However, some studies have shown that 5α-reductase inhibitor use increases the risk of developing diabetes mellitus, with an HR of 1.4 [13] and heart failure, with an increase of 0.3% [14]. Despite the widespread use of both drug classes, their comparative impact on the risk of recurrent IS remains insufficiently investigated.

Therefore, the primary aim of this study was to examine whether there is an association between pharmacological treatment for BPH and the risk of recurrent IS over time, comparing IS patients to those without BPH. By addressing this knowledge gap, we seek to inform clinical decisions regarding the treatment of BPH in patients who have previously experienced IS, ultimately helping to improve long-term outcomes for this vulnerable patient population.

## 2. Method

Since 1995, Taiwanese National Health Insurance has been a single-payer health care system organized by the Taiwanese government, covering 99% of the residents. The Longitudinal National Health Insurance Research Database (LNHIRD) is a representative database that includes data from 10% of the individuals covered by the Taiwanese National Health Insurance. The LNHIRD contains medical payment records for each visit or hospitalization. The claim dataset contained up to 3 International Classification of Diseases, Ninth Revision, Clinical Modification (ICD-9-CM) outpatient diagnosis codes and 5 ICD-9-CM inpatient diagnosis codes, visit dates, medication use, and medical costs [15]. In the present study, we used patients’ data, the LNHIRD between 2000 and 2015, to observe IS recurrence. Several studies have used the Taiwanese LNHIRD to identify clinical problems [8,9].

**Figure 1:**
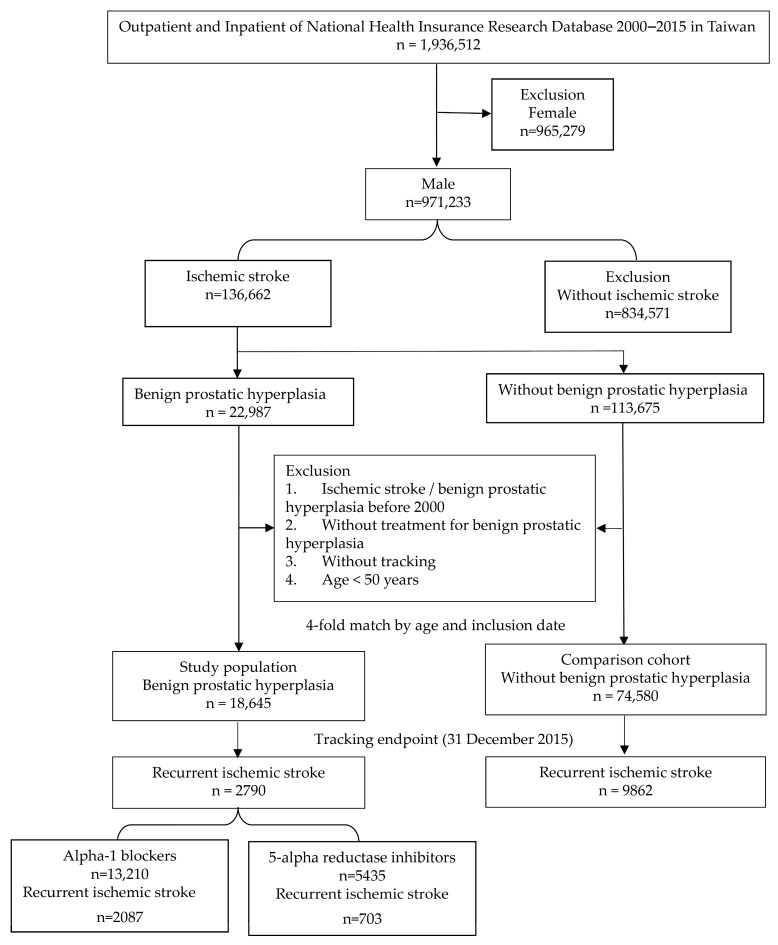
The flow-chart of this study.

This was a retrospective cohort study. Patients with newly diagnosed IS (ICD-9-CM: 433-437) and BPH (ICD-9-CM: 600) were identified from the database, and patients with BPH and first-ever IS diagnoses before 2000, those <50 years old, those not receiving BPH treatment drugs and those patients whose data were not tracked were excluded. The inclusion date was the date of the firstever IS with BPH treatment. The control group included IS patients without BPH who were matched 4 times by age and inclusion date and were followed up until 31 December 2015. The occurrence of an event was defined as a recurrent IS (ICD9-CM:433-437). This study was approved by the Tri-Services General Hospital Ethics Institutional Review Board (TSGHIRB E202516036). The flow-chart is shown in Figure 1.

BPH patients were treated with the following alpha-1 blocker drugs and corresponding Anatomical Therapeutic Chemical (ATC) codes: G04CA01 for alfuzosin; C02CA01 for prazosin; G04CA02 for tamsulosin; G04CA03 for terazosin; and C02CA04 for doxazosin. BPH patients were also treated with the following 5-alpha reductase inhibitors and corresponding ATC codes: G04CB01 for finasteride and G04CB02 for dutasteride.

Data on other comorbidities were extracted via the followingICD-9-CM codes: hypertension (401–405), diabetes (250), atrial fibrillation (427.31), hyperlipidemia (272), coronary artery disease (410–414), congestive heart failure (428), chronic kidney disease (580–589), peripheral arterial obstructive disease (443), chronic obstructive pulmonary disease (491, 492, 496), hypotension (458) and syncope (780.2). Antihypertensive medications and their corresponding ATC codes included C07 for beta blockers; C09A and C09B for angiotensin-converting enzyme inhibitors; C09C and C09D for angiotensin receptor blockers; C08 for calcium channel blockers; and C03 for diuretics. Antiplatelets corresponded to ATC codes included B01AC04-B01AC07, B01AC13, B01AC16, B01AC17, B01AC22, B01AC24, and B01AC25. Anticoagulants corresponded to ATC codes included B01AA, B01AB, B01AE, and B01AF. The revised Charlson comorbidity index (CCR_R) is the Charlson comorbidity index removed the following conditions: IS, hypertension, diabetes mellitus, atrial fibrillation, coronary artery disease, congestive heart failure, chronic kidney disease, peripheral obstructive disease, and chronic obstructive pulmonary disease.

### Statistical Analysis

Continuous variables are presented as the means ± standard deviations, and analyzed via Student’s *t* test. Categorical variables are presented as percentages and were compared via the Chi-square test. Cumulative incidence of recurrent IS was stratified by BPH and medication therapy using Kaplan-Meier curves with the log-rank test. The risk factors associated with long-term recurrent IS were evaluated by multivariate Cox proportional hazards regression after adjusting for the covariates and compared between IS patients receiving pharmacological treatment for BPH and BPH-free patients. The different defined daily dose and durations of pharmacological treatment were analyzed for the risk of recurrent IS. Death occurred in some patients with severe IS, and the risk of recurrent IS may be underestimated because of the competitive effect. For reducing underestimation of the risk, a competitive analysis was performed. *p* < 0.05 indicated statistical significance. All the data was analyzed via SPSS version 21 software (Asia Analytics Taiwan Ltd., Taipei, Taiwan).

## 3. Results

**Figure 2:**
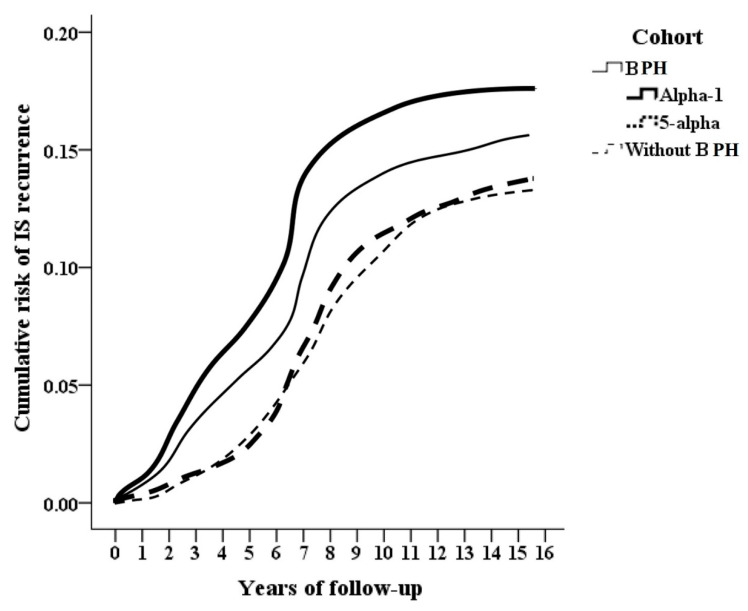
Cumulative incidence of recurrent ischemic stroke according log-rank test. Patients with benign prostatic hyperplasia had a greater risk of recurrent ischemic stroke (black, *p* < 0.001) and patients taking alpha-1 blockers had a greater risk (thick black, *p* < 0.001).

There were 18,645 IS patients with BPH, and 2790 (14.96%) developed recurrent IS; the control group without BPH included 74,580 patients, and 9862 (13.22%) developed recurrent IS. A total of 13,210 BPH patients were treated with alpha-1 blockers, and 2087 (15.8%) developed recurrent IS. A total of 5435 patients were treated with 5-alpha reductase inhibitors, and 703 (12.94%) developed recurrent IS (Figure 1). The cumulative incidence of recurrent IS is shown in Figure 2.

The patients with IS combined with BPH had higher rates of hypertension, diabetes, atrial fibrillation, coronary heart disease, heart failure, chronic renal disease, peripheral artery obstructive disease, chronic obstructive pulmonary disease, hypotension and syncope, and medical center visits (Table 1).

The risk factors for recurrent IS found an adjusted HR of 1.505 (95% CI: 1.112–1.829, *p* < 0.001) for patients with BPH, 1.577 (95% CI: 1.286–1.932, *p* < 0.001) for older individuals (≥65 years old), 2.068 (95% CI: 1.486–2.656, *p* < 0.001) for individuals with hypertension, 1.67 (95% CI: 1.286–2.043, *p* < 0.001) for individuals with diabetes, 2.127 (95% CI: 1.654–2.586, *p* < 0.001) for individuals with atrial fibrillation, 1.337 (95% CI: 1.054–1.73, *p* = 0.023) for individuals with hyperlipidemia, 2.157 (95% CI: 1.811–2.489, *p* < 0.001) for individuals with coronary artery disease, 2.586 (95% CI: 2.163–3.068, *p* < 0.001) for individuals with congestive heart failure, 1.67 (95% CI: 1.193–2.266, *p* < 0.001) for individuals with chronic kidney disease, 1.549 (95% CI: 1.111–2.041, *p* < 0.001) for individuals with peripheral arterial obstructive disease, 1.308 (95% CI: 1.169–1.389, *p* < 0.001) for individuals with disease severity CCI_R, 1.45 (95% CI: 1.173–1.728, *p* < 0.001) for individuals diagnosed during winter (versus spring), 2.065 (95% CI: 1.615–2.95, *p* < 0.001) for individuals living in the highest degree of urbanization, 1.481 (95% CI: 1.086–1.97, *p* = 0.007) for individuals living in the second-highest urbanization level, 2.007 (95% CI: 1.199–2.825, *p* < 0.001) for individuals being treated in medical centers (versus local hospitals), and 1.751 (95% CI: 1.076–2.568, *p* = 0.012) for individuals being treated in regional hospitals (Table 2).

Older patients and patients with hypertension receiving 5-alpha reductase inhibitors were compared with patients who received alpha-1 blockers (Supplementary Table S1,the baseline characteristics of benign prostatic hyperplasia patients who received different medications). There was no significant difference in the risk of recurrent IS between patients who received 5-alpha reductase inhibitors and alpha-1 blockers (Supplementary Table S2, factors of recurrent ischemic stroke among different medicines in patients with benign prostatic hyperplasia according to Cox regression).

A competing risk model showed an adjusted HR of 1.544 (95% CI: 1.128–1.896, *p* < 0.001) in BPH patients. The adjusted HRs for the risk of IS recurrence in the pharmacological treatment of patients with BPH were as follows: an adjusted HR of 1.581 (95% CI: 1.16–1.915, *p* < 0.001) for individuals receiving alpha-1 blocker treatment (versus no BPH), and an adjusted HR of 1.219 (95% CI: 0.921–1.569, *p* = 0.089) for individuals receiving 5-alpha reductase inhibitor treatment (versus no BPH) after adjusting for the other covariates shown in Table 3. BPH patients who received more than one third of their alpha-1 blockers defined daily dose (DDD) for 1 month were at risk of recurrent IS. BPH patients who received 5-alpha reductase inhibitors for more than two thirds of the DDD for 6 months had a risk of recurrent IS (Table 3).

## 4. Discussion

This study revealed that first-ever IS patients with concurrent BPH were more likely to develop recurrent IS over time. Among patients with BPH, those using pharmacological treatment had an increased risk of recurrent IS. Elderly individuals and those with other arteriosclerotic factors were more likely to develop recurrent IS. When patients develop IS, appropriate treatment is needed to prevent recurrent IS. For patients with BPH, medication should be used more carefully to reduce the incidence of recurrent IS.

Baroreflex sensitivity and parasympathetic activity decrease in both the acute and chronic phases of IS. Sympathetic activity induces cerebral artery constriction, and parasympathetic activity induces cerebral artery dilatation [5]. Patients with acute IS have disrupted dynamic cerebral autoregulation (dCA) [16,17]. The reduction in dCA after IS is related to the recurrent IS after pharmacological treatment of patients with BPH.

Moreover, BPH is frequently accompanied by nocturia, which disrupts sleep, thereby increasing sympathetic nervous activity and fostering chronic inflammation and metabolic syndrome, conditions that increase the risk of cardiovascular morbidity [18,19]. Previous studies have shown that BPH is associated with an increased burden of atherosclerosis and endothelial dysfunction, and patients with severe lower urinary tract symptoms have a 1.7-fold increased likelihood of experiencing MACEs [12]. In Japan, studies have revealed increases in the risks of angina, stroke, and atrial fibrillation among patients with BPH, although the incidence of myocardial infarction has remained unaffected [7]. Our study revealed an increased risk of recurrent IS in patients with IS and BPH after long-term follow-up.

Although alpha-1 blockers are useful for alleviating obstructive urinary symptoms and enhancing quality of life [11], they cannot prevent disease progression, they influence cerebral autoregulation [20] and they increase the propensity of the patient to develop hypotension with standing intolerance [21,22]. Among individuals with chronic hypertension, improvements in cerebral hemodynamics, such as parasympathetic cerebral vessel dilation, were detected within 1–2 months following the initiation of alpha-blocker therapy [23]. Our study revealed that the use of alpha-1 blockers used for more than 1 month resulted in a higher risk of recurrent IS. In contrast, 5α-reductase inhibitors, which diminish prostatic volume without perturbing blood pressure, present a more auspicious alternative for mitigating the risk of recurrent IS without affecting hemodynamics or cerebral perfusion [10]. Our study revealed that the use of 5α-reductase inhibitors for less than 6 months was not associated with risk in patients with recurrent IS. However, the IS patients with BPH who take high doses of 5α-reductase inhibitors over a long period of time are at risk of recurrent IS, which is potentially due to a greater prostate volume and greater risk of diabetes mellitus or congestive heart failure [13,14].

There was an increased risk of hospitalization or emergency room evaluation for hypotension, with an odds ratio (OR) of 1.8, in patients treated with alpha-1 antagonists within three months. Older age, hypertension and a systolic blood pressure greater than 140 mmHg are associated with an increased risk of developing orthostatic hypotension [24]. A previous study revealed that IS occurs in the first 3 weeks after the use of alpha-1 blockers, and is potentially related to hypotension [8]. Approximately 70% of the BPH population used alpha-1 blockers in our study, resulting in more patients suffering from hypotension and syncope. Although the crude HR for the risk of recurrent IS was 1.7 for hypotension, after adjusting for other risks, the HR (1.5) was non-significant. This finding suggests that hypotension has a certain trend effect; however, atherosclerosis-related risk factors and BPH risk factors should be further considered. A previous meta-analysis revealed that patients taking any type of blood pressure-lowering medication subsequently had lower rates of recurrent IS than patients taking a placebo did, with an OR of 0.8 [25]. However, our study revealed that the use of antihypertensive drugs was associated with a greater risk of recurrent IS with a crude HR of 1.865. The potential reasons are that the control rate of hypertension in males in Taiwan is only 56.7% [26], or that antihypertensive agents combined with alpha-1 blockers potentially lead to the development of hypotension.

The independent risk factors for recurrent IS included older age, with an HR of 1.6, and our study showed similar results. The typical risk factors were the following: hyperlipidemia, with an HR of 2.22; hypertension, with an HR of 2; and ischemic heart disease, with an HR of 2.1 [27]. Our study revealed similar risks of developing hypertension and coronary artery disease. The risk of developing hyperlipidemia was lower, possibly because the claims dataset was coded for the use of antilipidemic agent treatment. A meta-analysis revealed a greater risk of recurrent IS among patients with diabetes mellitus, with an HR of 1.5 [28], and our study showed similar results. Patients with atrial fibrillation carry a risk of recurrent stroke, with an HR of 1.54 in the UK [29]. Our study revealed a greater risk of atrial fibrillation with a lower anticoagulant therapy usage rate [30]. A previous study revealed that heart failure status was independently associated with three-month recurrence of IS, with an OR of 2.4 [31]. Our study revealed similar results. The univariate OR for IS was 1.8 in patients with a lower eGFR [32], which was associated with a lower risk of chronic kidney disease after adjusting for other atherosclerosis factors in our study. Peripheral obstructive artery disease related to intracranial carotid atherosclerosis had an OR of 1.4 [33]. Our study revealed similar results.

Cold weather can easily cause vasoconstriction and recurrent IS [34]. Patients living in more urbanized areas have poorer lifestyles and eating habits, exercise less and are more likely to develop recurrent IS. Compared with the treatment of patients admitted to local hospitals, that of patients with recurrent IS is more complicated; thus, they are often sent to medical centers and regional hospitals for management and have a greater risk of recurrent IS. A greater severity of disease after excluding other preexisting risk factors increases the risk of recurrent IS. The use of antiplatelets after IS reduces the risk of recurrent IS, with an HR of 0.6, and the majority of patients with IS in Taiwan receive antiplatelets for secondary prevention [30].

The strength of this study is the discovery that the long-term incidence of recurrent IS is related to BPH status among Asian men. For patients with both IS and BPH, low-dose and short-term alpha-1 blocker treatment and high-dose and long-term 5α-reductase inhibitors treatment were associated with an increased risk of recurrent IS. The operation for BPH may be good choice in IS patients; the clinicians need to carefully order the medications for patients with IS.

This study has several limitations. First, comprehensive data concerning lifestyle factors such as tobacco usage, alcohol consumption, or physical activity, as well as target management of hypertension, glucose levels, or lipid profiles in the claims database, which may modulate the risk of recurrent IS, were not provided. Second, the absence of imaging data, such as cortical lesions, large-vessel classification or intracranial atherosclerosis, is associated with the recurrence of IS. The presence of white matter hyperintensity is related to the one-year recurrence of IS, with an HR of 1.03 in Korea [35]. Third, BPH without medication was excluded; the operation of BPH for recurrent IS needs to study in the future. Finally, while hypotension did appear to trend towards an increased risk of recurrent IS, definitive causality could not be ascertained. Further prospective investigations are needed to elucidate the roles of baroreflex sensitivity and autonomic dysfunction in this regard.

## 5. Conclusions

Patients with IS having BPH who underwent medication treatment had a greater risk of long-term recurrent IS than patients without BPH. A higher risk of recurrent IS was noted for patients that were treated with low-dose and short-term alpha-1 blocker treatment and high-dose BPH medications of 5-αreductase inhibitors over a long period of time. The risk factors found in this study must be considered and tailored to the patient’s condition to achieve optimal therapeutic effects and prevent the recurrence of IS.

## Figures and Tables

**Table 1:** Baseline characteristics of the patients.

Variables	Total n = 93,225	Benign Prostate Hyperplasia n =18,645	Benign Prostate Hyperplasia Free n =74,580	*p*
Age (years old)	70.71 ± 18.80	70.69 ± 18.76	70.72 ± 18.81	0.846
Age groups (years old)				0.999
50–64	31,295 (33.57%)	6259 (33.57%)	25,036 (33.57%)	
≥65	61,930 (66.43%)	12,386 (66.43%)	49,544 (66.43%)	
Hypertension	38,473 (41.27%)	8015 (42.99%)	30,458 (40.84%)	<0.001 *
Antihypertensive agents	42,841 (45.95%)	8971 (48.11%)	33,870 (45.41%)	<0.001 *
Diabetes mellitus	40,379 (43.31%)	8275 (44.38%)	32,104 (43.05%)	0.001 *
Atrial fibrillation	13,097 (14.05%)	3032 (16.26%)	10,065 (13.50%)	<0.001 *
Hyperlipidemia	35,449 (38.03%)	7389 (39.63%)	28,060 (37.62%)	<0.001 *
Coronary artery disease	33,325 (35.75%)	6845 (36.71%)	26,480 (35.51%)	0.002 *
Congestive heart failure	18,400 (16.48%)	3892 (17.27%)	14,508 (16.29%)	<0.001 *
Chronic kidney disease	32,974 (35.37%)	6986 (37.47%)	25,988 (34.85%)	<0.001 *
Peripheral artery obstructive disease	30,437 (32.65%)	6425 (34.46%)	24,012 (32.20%)	<0.001 *
Chronic obstructive pulmonary disease	20,154 (21.62%)	4987 (26.75%)	15,167 (20.34%)	<0.001 *
Hypotension	13,242 (14.20%)	2870 (15.39%)	10,372 (13.91%)	<0.001 *
Syncope	5402 (5.79%)	1298 (6.96%)	4104 (5.50%)	<0.001 *
Antiplatelets	8723 (9.36%)	1752 (9.4%)	6971 (9.35%)	0.835
Anticoagulants	5233 (5.61%)	1023 (5.49%)	4210 (5.64%)	0.401
CCI_R	0.81 ± 1.09	0.84 ± 1.12	0.80 ± 1.08	<0.001 *
Season				0.887
Spring (Mar–May)	23,864 (25.60%)	4760 (25.53%)	19,104 (25.62%)	
Summer (Jun–Aug)	24,548 (26.33%)	4876 (26.15%)	19,672 (26.38%)	
Autumn (Sep–Nov)	22,976 (24.65%)	4623 (24.79%)	18,353 (24.61%)	
Winter (Dec–Feb)	21,837 (23.42%)	4386 (23.52%)	17,451 (23.40%)	
Location				<0.001 *
Northern Taiwan	28,484 (30.55%)	5689 (30.51%)	22,795 (30.56%)	
Central Taiwan	25,898 (27.78%)	5331 (28.59%)	20,567 (27.58%)	
Southern Taiwan	24,079 (25.83%)	5245 (28.13%)	18,834 (25.25%)	
Eastern Taiwan	13,199 (14.16%)	2026 (10.87%)	11,173 (14.98%)	
Outlets islands	1565 (1.68%)	354 (1.90%)	1211 (1.62%)	
Urbanization level				<0.001 *
1 (The highest)	25,450 (27.30%)	5298 (28.42%)	20,152 (27.02%)	
2	30,375 (32.58%)	5786 (31.03%)	24,589 (32.97%)	
3	16,269 (17.45%)	3124 (16.76%)	13,145 (17.63%)	
4 (The lowest)	21,131 (22.67%)	4437 (23.80%)	16,694 (22.38%)	
Level of care				<0.001 *
Medical center	30,425 (32.64%)	7342 (39.38%)	23,083 (30.95%)	
Regional hospital	34,285 (36.78%)	6312 (33.85%)	27,973 (37.51%)	
Local hospital	28,515 (30.59%)	4991 (26.77%)	23,524 (31.54%)	

* *p* < 0.05.

**Table 2:** The risk factors of recurrent ischemic stroke.

Risk Factors	Adjusted Hazard Ratio	*p*
Benign prostate hyperplasia	1.505 (95% C.I.: 1.112–1.829)	<0.001 *
Age group (years-old)		
≥65	1.577 (95% C.I.: 1.286–1.932)	<0.001 *
Hypertension	2.068 (95% C.I.: 1.486–2.656)	<0.001 *
Diabetes mellitus	1.67 (95% C.I.: 1.286–2.043)	<0.001 *
Atrial fibrillation	2.127 (95% C.I.: 1.654–2.586)	<0.001 *
Hyperlipidemia	1.337 (95% C.I.: 1.054–1.73)	0.023 *
Coronary artery disease	2.157 (95% C.I.: 1.811–2.489)	<0.001 *
Congestive heart failure	2.586 (95% C.I.: 2.163–3.068)	<0.001 *
Chronic kidney disease	1.67 (95% C.I.: 1.193–2.266)	<0.001 *
Peripheral artery obstructive disease	1.549 (95% C.I.: 1.111–2.041)	<0.001 *
Chronic obstructive pulmonary disease	1.315 (95% C.I.: 0.996–1.707)	0.057
Hypotension	1.537 (95% C.I.: 0.917–1.891)	0.094
Syncope	1.12 (95% C.I.: 0.754–1.738)	0.249
Antiplatelets	1.38 (95% C.I.: 0.793–2.888)	0.207
Anticoagulants	1.134 (95% C.I.: 0.611–2.606)	0.398
CCI_R	1.308 (95% C.I.: 1.169–1.389)	<0.001 *
Season		
Spring	Reference	
Summer	0.953 (95% C.I.: 0.714–1.137)	0.286
Autumn	1.199 (95% C.I.: 0.986–1.428)	0.064
Winter	1.45 (95% C.I.: 1.173–1.728)	<0.001 *
Location	Multicollinearity with urbanization level	
Urbanization levels		
1 (The highest)	2.065 (95% C.I.: 1.615–2.95)	<0.001 *
2	1.481 (95% C.I.: 1.086–1.97)	0.007 *
3	1.146 (95% C.I.: 0.859–1.417)	0.148
4 (The lowest)	Reference	
Hospital levels		
Medical center	2.007 (95% C.I.: 1.199–2.825)	<0.001 *
Regional hospital	1.751 (95% C.I.: 1.076–2.568)	0.012 *
Local hospital	Reference	

* *p* < 0.05.

**Table 3:** Factors associated with the recurrence of ischemic stroke among different subgroups of patients with benign prostatic hyperplasia according to Cox regression and Bonferroni correction for multiple comparisons.

Subgroups	Population	Events	Person–Years	Rate (/per 1000)	Adjusted Hazard Ratio	*p*
BPH-free	74,580	9862	646,608.57	15.25	Reference	
BPH with medication	18,645	2790	161,092.83	17.32	1.505 (95% CI: 1.112–1.829)	<0.001 *
Competing risk					1.544 (95% CI: 1.128–1.896)	<0.001 *
Alpha-1 blockers	13,210	2087	114,135.26	18.29	1.581 (95% CI:1.16–1.915)	<0.001 *
DDD < 33%	4320	580	37,325.22	15.54	1.35 (95% CI:0.997–1.64)	0.052
DDD (33–67%)	5796	911	50,079.45	18.19	1.581 (95% CI: 1.168–1.921)	<0.001 *
DDD ≥ 67%	3094	596	26,730.59	22.3	1.938 (95% CI:1.432–2.355)	<0.001 *
≤1 month	5001	650	43,209.70	15.04	1.305 (95% CI:0.964–1.589)	0.084
1 month–6 months	5846	980	50,513.24	19.4	1.685 (95% CI:1.24–2.046)	<0.001 *
>6 months	2363	457	20,412.32	22.39	1.946 (95% CI:1.437–2.364)	<0.001 *
5-alpha reductase inhibitors	5435	703	46,957.57	14.97	1.219(95%CI:0.921–1.569)	0.089
DDD < 33%	1032	114	8918.34	12.78	1.111 (95% CI: 0.818–1.35)	0.191
DDD (33–67%)	2795	367	24,186.01	15.17	1.32 (95% CI:0.977–1.602)	0.078
DDD ≥ 67%	1608	222	13,853.22	16.03	1.394 (95% CI:1.029–1.692)	0.035 *
≤1 month	1203	143	10,397.52	13.75	1.195 (95% CI:0.875–1.433)	0.134
1 month–6 months	2965	386	25,670.28	15.04	1.308 (95% CI:0.966–1.589)	0.082
>6 months	1267	174	10,889.77	15.98	1.391 (95% CI: 1.025–1.732)	0.038 *

* *p* < 0.05; BPH: benign prostatic hyperplasia; CI: confidence interval; DDD: defined daily dose; all the analyses were adjusted for age, comorbidities, season, location, urbanization levels and level of health care.

## Data Availability

Restrictions apply to the availability of this data. Data was obtained from the National Health Insurance database and is available from the authors with the permission of the National Health Insurance Administration of Taiwan. Because Taiwan’s National Health Insurance database allows researchers access to research centers for analysis, it only provides the analyzed data. Original data can only be provided with the data center’s permission.

## Supplementary Materials

The following supporting information can be downloaded at: https://www.mdpi.com/article/10.3390/life16020311/s1, Supplementary Table S1, the baseline characteristics of benign prostatic hyperplasia patients who received different medications. Supplementary Table S2, factors of recurrent ischemic stroke among different medicines in patients with benign prostatic hyperplasia according to Cox regression.
