# Supplementary material for: Increased Risk of Recurrent Ischemic Stroke in Male Patients Taking Medications for Benign Prostatic Hyperplasia
Source: Life (Basel). 2026 Feb 11;16(2):311. doi: 10.3390/life16020311 (PMC12942141; doi:10.3390/life16020311)
Supplement: Supplementary file 1 [file life-16-00311-s001.zip › life-4084421-supplementary-proof done v2 1.pdf]

Supplementary table 1: The baseline characteristics of benign prostatic hyperplasia patients who received different medications

Variables	BPH with medications 18,645	Alpha-1 blocker 13,210	5-alpha reductase inhibitor 5435	*p*
Age (years)	70.69 ± 18.76	70.45 ± 18.71	71.27 ± 18.87	0.006*
Age groups (years)				0.746
50 - 64	6259 (33.57%)	4444 (33.64%)	1815 (33.39%)	
≥ 65	12,386 (66.43%)	8766 (66.36%)	3620 (66.61%)	
Hypertension	8015 (42.99%)	5618 (42.53%)	2397 (44.1%)	0.048*
Antihypertensive agents	8971 (48.11%)	6379 (48.29%)	2592 (47.69%)	0.457
Diabetes mellitus	8275 (44.38%)	5885 (44.55%)	2390 (43.97%)	0.472
Atrial fibrillation	3032 (16.26%)	2112 (15.99%)	920 (16.93%)	0.114
**Hyperlipidemia**	7389 (39.63%)	5224 (39.55%)	2165 (39.83%)	0.714
Coronary artery disease	6845 (36.71%)	4865 (36.83%)	1980 (36.43%)	0.609
Congestive heart failure	3892 (17.27%)	2277 (17.24%)	1615 (17.32%)	0.878
Chronic kidney disease	6986 (37.47%)	4925 (37.28%)	2061 (37.92%)	0.413
PAOD	6425 (34.46%)	4533 (34.31%)	1892 (34.81%)	0.517
COPD	4987 (26.75%)	3526 (26.69%)	1461 (26.88%)	0.791
Hypotension	2870 (15.39%)	2022 (15.31%)	848 (15.6%)	0.611
Syncope	1298 (6.96%)	930 (7.04%)	368 (6.77%)	0.512
Antiplatelets	1752 (9.4%)	1259 (9.53%)	493 (9.07%)	0.328
Anticoagulants	1023 (5.49%)	718 (5.44%)	305 (5.61%)	0.631
CCI_R	0.84 ± 1.12	0.80 ± 1.10	0.94 ± 1.16	< 0.001*
Season				0.994
Spring (Mar - May)	4760 (25.53%)	3379 (25.58%)	1381 (25.41%)	
Summer (Jun - Aug)	4876 (26.15%)	3452 (26.13%)	1424 (26.2%)	
Autumn (Sep - Nov)	4623 (24.79%)	3270 (24.75%)	1353 (24.89%)	
Winter (Dec - Feb)	4386 (23.52%)	3109 (23.54%)	1277 (23.5%)	
Location				0.799
Northern Taiwan	5689 (30.51%)	4037 (30.56%)	1652 (30.4%)	
Middle Taiwan	5331 (28.59%)	3779 (28.61%)	1552 (28.56%)	
Southern Taiwan	5245 (28.13%)	3716 (28.13%)	1529 (28.13%)	

Eastern Taiwan	2026 (10.87%)	1438 (10.89%)	588 (10.82%)	
Outlets islands	354 (1.9%)	240 (1.82%)	114 (2.1%)	
Urbanization level				0.529
1 (The highest)	5298 (28.42%)	3746 (28.36%)	1552 (28.56%)	
2	5786 (31.03%)	4072 (30.83%)	1714 (31.54%)	
3	3124 (16.76%)	2211 (16.74%)	913 (16.8%)	
4 (The lowest)	4437 (23.8%)	3181 (24.08%)	1256 (23.11%)	
Level of care				0.879
Medical center	7342 (39.38%)	5198 (39.35%)	2144 (39.45%)	
Regional hospital	6312 (33.85%)	4486 (33.96%)	1826 (33.6%)	
Local hospital	4991 (26.77%)	3526 (26.69%)	1465 (26.95%)	

*$p<0.05$; PAOD: peripheral artery obstructive disease; COPD: chronic obstructive pulmonary disease

Table S2. Factors of recurrent ischemic stroke among different medicines in patients with benign prostatic hyperplasia according to Cox regression

Variables	Adjusted HR (95% confidence interval)	*p*
BPH medicine		
Alpha-1 blocker	Reference	
5-alpha reductase inhibitor	1.297 (0.498 – 2.301)	0.507
Age group (years)		
50 - 64	Reference	
≥ 65	1.604 (1.32 – 1.939)	< 0.001*
Hypertension	2.104 (1.525 – 2.666)	< 0.001*
Antihypertensive agents	1.605 (1.104 – 2.049)	< 0.001*
Diabetes mellitus	1.699 (1.32 – 2.051)	< 0.001*
Atrial fibrillation	2.165 (1.698 – 2.595)	< 0.001*
Hyperlipidemia	1.361 (1.082 – 1.737)	0.009*
Coronary artery disease	2.195 (1.859 – 2.498)	< 0.001
Congestive heart failure	2.632 (2.22 – 3.08)	< 0.001*
Chronic kidney disease	1.699 (1.225 – 2.274)	< 0.001*
Peripheral artery obstructive disease	1.577 (1.141 – 2.049)	< 0.001*
Chronic obstructive pulmonary disease	1.338 (1.023 – 1.713)	0.039*
Hypotension	1.564 (0.942 – 1.898)	0.076
Syncope	1.14 (0.774 – 1.744)	0.301
Antiplatelets	1.383 (0.793 – 2.888)	0.584
Anticoagulants	1.136 (0.611 – 2.606)	0.677
CCI_R	1.331 (1.2 – 1.395)	< 0.001*
Season		
Spring	Reference	
Summer	0.97 (0.733 – 1.142)	0.265
Autumn	1.22 (1.013 – 1.433)	0.043*
Winter	1.476 (1.204 – 1.735)	< 0.001*
Urbanization level		
1 (The highest)	2.101 (1.658 – 2.961)	< 0.001*
2	1.507 (1.115 – 1.977)	< 0.001*
3	1.166 (0.882 – 1.423)	0.129
4 (The lowest)	Reference	
Level of care		

Medical center	2.043 (1.231 – 2.836)	< 0.001*
Regional hospital	1.782 (1.105 – 2.578)	< 0.001*
Local hospital	Reference	

*p<0.05; HR: hazard ratio
